# Responsible AI readiness in a humanitarian and development ecosystem: an exploratory case study

**DOI:** 10.3389/fpubh.2026.1937518

**Published:** 2026-09-01

**Authors:** Yasmine Attia, Diana Maddah, Al-Anoud Al-Kaabi, Ghalia Seifo, Mohammed Bader Al-Sada, Jesha Mohammed Ali Mundodan, Abdelaziz Khatir Jadeed, Niveen M. E. Abu-Rmeileh, Kwalombota Kwalombota

**Affiliations:** 1 Department of Public Health, College of Health Sciences, Qatar University, Doha, Qatar; 2 Qatar Fund for Development (QFFD), Doha, Qatar; 3 United Nations Office for the Coordination of Humanitarian Affairs (UN OCHA), Doha, Qatar; 4 Qatar Red Crescent Society (QRCS), Doha, Qatar; 5 Ministry of Public Health (MoPH), Doha, Qatar; 6 Qatar Charity (QC), Doha, Qatar; 7 Foreign, Commonwealth and Development Office (FCDO), London, United Kingdom

**Keywords:** artificial intelligence, data responsibility, humanitarian, organizational readiness, Qatar, technology acceptance

## Abstract

Humanitarian and development organizations are increasingly exploring artificial intelligence (AI) to support information-intensive work, yet responsible adoption depends on both staff readiness and organizational enabling conditions. This study provides an exploratory stakeholder-based snapshot of AI readiness within Qatar’s humanitarian and development ecosystem. A cross-sectional, bilingual online survey was administered to humanitarian-sector stakeholders (*N* = 83) using purposive and snowball recruitment initiated through a multi-stakeholder network. Readiness was examined using a dual conceptual lens: the Unified Theory of Acceptance and Use of Technology (UTAUT) was used to interpret adoption-related perceptions, and the Organizational Digital Transformation Readiness (ODTR) model was used to structure organizational readiness conditions. Descriptive indicators were summarized, and composite indices were calculated on a 0–100 scale. Adoption-related perceptions were high in this sample (UTAUT-informed index mean: 90.8/100), whereas organizational readiness conditions were more moderate and variable (ODTR index mean: 56.8/100). Governance and safeguards represented the weakest organizational domain (mean: 43.6/100). The most frequently reported medium or major barriers were insufficient staff skills to work with data or AI tools (89.2%), technical complexity or difficulty using AI tools (86.7%), and concerns about data privacy or potential misuse (84.3%). Overall, the findings suggest a layered readiness pattern in which individual openness to AI appears stronger than the visibility and consistency of organizational governance and capability conditions. These results should be interpreted as descriptive and context-specific rather than representative of the wider sector. Even so, they provide an empirical baseline that may help inform future efforts to strengthen governance, safeguards, and capacity-building for responsible AI adoption in humanitarian and development settings.

## 1 Introduction

Humanitarian organizations operate across a wide range of contexts where people face heightened vulnerability and urgent needs, including armed conflict, sudden-onset disasters, climate-related shocks, public health emergencies, displacement, and complex socio-economic crises (1, 2). In these settings, organizations often make high-stakes decisions under time pressure, fragmented information, and constrained resources (3). Importantly, humanitarian action is not limited to contexts experiencing active conflict; it is also enabled by networks of organizations that finance, coordinate, deliver, and support preparedness and response across borders (4, 5). Humanitarian needs are growing in scale and complexity while funding remains limited, increasing pressure on organizations to deliver assistance efficiently and accountably (6–9).

in this context, artificial intelligence (AI), including machine learning and increasingly generative AI, has become more accessible through widely available tools that can support information processing, translation, summarization, pattern recognition, and decision support (10). Humanitarian coordination bodies and practitioners have highlighted potential applications such as improved situational awareness, faster analysis of needs and contextual trends, enhanced operational planning, and streamlined internal workflows (11). However, implementing AI in field settings can also introduce operational risks when data are incomplete, context changes rapidly, or safeguards are unclear. In high-stakes environments, errors or biased outputs can affect decisions and service delivery, reinforcing the importance of responsible use (12). Evidence reviews and practitioner-oriented resources describe a range of potential uses – such as early warning and risk analysis, crisis mapping, translation and communication support, and administrative efficiencies – while emphasizing that real-world benefit depends on data quality, operational context, and implementation choices (13, 14). Interest in AI is not limited to operational efficiency; it is also linked to broader goals around modernization, governance, and sustainable service delivery. At the organizational level, AI’s near-term value is often framed around augmenting staff work rather than replacing decision-making, which is particularly relevant in settings where accountability and duty of care are high (15–17). Humanitarian organizations have responded by articulating responsible AI positions grounded in humanitarian principles and institutional mandates (18). For example, the International Committee of the Red Cross (ICRC) has published an AI policy intended to support safe exploration of AI use, explicitly anchored in humanitarian principles and operational realities (19).

Practical guidance increasingly emphasizes structured adoption processes across the AI lifecycle, including problem definition, data readiness, governance, monitoring, and evaluation (20). Within such ecosystems, readiness to adopt AI responsibly is relevant because decisions about tools, governance, capacity-building, and data practice may influence both locally managed programming and internationally supported operations (21). However, AI introduces widely discussed risks in humanitarian contexts, including threats to privacy and data protection, biased outputs that may worsen inequities, limited transparency that complicates accountability, and potential erosion of trust among affected communities (12, 21).

This risk is compounded by the rapid emergence of “shadow AI” unauthorized personal use of commercial tools – which is already widespread. The January 2026 global pulse survey found that while 95.3% of humanitarian professionals have used AI tools for work and 75.0% use them daily or weekly, organizational integration remains extremely low at only 9.0%, creating a widening gap between individual uptake and institutional safeguards (22). This includes guidance emphasizing proportionality, precaution, and ‘do no harm’ when exploring and deploying AI-enabled tools (22). These developments underscore that readiness must include ethical and governance maturity, not only technical capability. Because many AI applications rely on sensitive personal and operational data, readiness is also closely tied to data responsibility. The Inter-Agency Standing Committee (IASC) Operational Guidance on Data Responsibility in Humanitarian Action (2023) and OCHA’s Data Responsibility Guidelines (2025) provide principles, processes, and tools for managing data safely and ethically, implying the need for clear policies, defined roles and accountability, risk assessment processes, and staff understanding of responsible data use (23, 24).

Taken together, the literature presents a tension between AI’s anticipated operational value and the institutional conditions required for its responsible use. Application-oriented studies emphasize improvements in situational awareness, humanitarian mapping, translation, information processing, and decision support (11, 13, 14), whereas governance-focused scholarship and organizational guidance emphasize risks involving privacy, bias, transparency, accountability, and community trust (12, 19, 20). The evidence also reveals a disconnect between individual experimentation and formal organizational adoption: recent survey findings indicate widespread use of AI by humanitarian professionals but substantially lower levels of institutional integration (22). Much of the existing evidence nevertheless remains conceptual, policy-oriented, or focused on particular applications rather than empirically assessing whether organizations possess the governance, technical resources, workforce capabilities, and internal culture required for responsible implementation. This unresolved gap makes it difficult to determine whether growing enthusiasm for AI reflects sustainable organizational readiness or adoption occurring ahead of adequate safeguards.

In Qatar, the humanitarian and development ecosystem includes national and international actors as well as institutions that finance, coordinate, or partner in humanitarian interventions abroad, including some Qatar-based organizations that support or implement field operations internationally (25, 26). These actors contribute not only to development programming but also to preparedness, emergency response support, and recovery-oriented assistance across multiple crisis contexts (26). This aligns directly with Qatar National Vision 2030, the Third National Development Strategy (2024–2030), and the Qatar Digital Agenda 2030, which position digital transformation and AI as key enablers of government excellence, sustainable development, efficient service delivery, and a knowledge-based economy (27). In this context, interest in AI is not limited to operational efficiency; it is also linked to broader goals around modernization, governance, and sustainable service delivery. Within such ecosystems, readiness to adopt AI responsibly is relevant because decisions about tools, governance, capacity-building, and data practice may influence both locally managed programming and internationally supported operations (28). For example, the Qatar Fund for Development describes its role as connecting state institutions, humanitarian organizations, and United Nations (UN) agencies to support development and humanitarian interventions, and UNHCR identifies QFFD as a donor partner supporting vulnerable populations across multiple contexts (29). However, there is limited empirical evidence on how prepared institutions, especially within specific national humanitarian ecosystems are, to adopt AI responsibly in practice.

To assess readiness systematically, a dual conceptual lens linking organizational readiness conditions with adoption-related perceptions was adopted. Organizational readiness conditions were structured using the Organizational Digital Transformation Readiness (ODTR) model, while individual adoption-related perceptions were interpreted using the Unified Theory of Acceptance and Use of Technology (UTAUT) (30, 31). Together, these frameworks connect organizational enablers (e.g., governance, capacity, and digital foundations) with staff perceptions that shape whether AI is adopted in day-to-day practice (30, 31).

Previous research and humanitarian guidance have primarily examined potential AI applications, ethical and data-protection risks, responsible-use principles, or broader organizational digital transformation. However, empirical studies have rarely examined whether positive individual perceptions of AI are accompanied by the organizational governance, safeguards, digital foundations, and workforce capabilities required for responsible implementation, particularly within a specific national humanitarian and development ecosystem (32, 33). Consequently, there remains limited evidence on how individual adoption-related readiness and organizational readiness conditions interact in practice. The present study addresses this gap by integrating UTAUT-informed adoption perceptions with ODTR-based organizational readiness conditions to provide a structured assessment of responsible AI readiness among stakeholders in Qatar’s humanitarian and development ecosystem. This dual-level approach enables readiness to be examined as a layered construct rather than as a single organizational attribute.

Accordingly, this paper reports findings from an exploratory cross-sectional survey of humanitarian-sector stakeholders in Qatar, focusing on (i) current digital and data practices, (ii) perceived organizational readiness conditions and barriers related to AI adoption, and (iii) staff perceptions and self-reported readiness to use AI. Rather than providing a representative sector-wide assessment, the study offers a descriptive snapshot intended to inform early discussion and future assessment of responsible AI readiness in this national ecosystem.

## 2 Methods

### 2.1 Study design

A cross-sectional survey was conducted to provide a baseline assessment of AI readiness among professionals working within Qatar’s humanitarian and development ecosystem.

### 2.2 Survey instrument

The survey questionnaire was developed through an iterative, multi-stage process to ensure its validity and relevance to the local context. The initial item pool was informed by a review of existing literature and humanitarian-sector guidance on responsible data and AI (e.g., IASC and OCHA data responsibility guidance and the ICRC AI policy), and was structured using the conceptual domains of the Organizational Digital Transformation Readiness and Unified Theory of Acceptance and Use of Technology frameworks (23, 24, 30, 34). UTAUT was used as an interpretive framework rather than implemented as a full validated multi-item instrument. Given the exploratory purpose of the study and the need to keep the questionnaire feasible for busy humanitarian professionals, selected items were treated as proxy indicators for conceptually related UTAUT constructs. These proxy measures are reported descriptively and should be interpreted as indicative rather than as validated scale scores. The draft instrument then went through the following steps to support validity and contextual relevance.

#### 2.2.1 Stakeholder consultation

The questionnaire was reviewed over several consultative rounds with a multi-stakeholder Technical Working Group (TWG). This group, composed of senior representatives from various Qatar-based humanitarian organizations, provided critical feedback to ensure the questions were clear, contextually appropriate, and relevant to operational realities.

#### 2.2.2 Expert review

Following stakeholder consultations, the revised draft was reviewed by external subject-matter experts in humanitarian technology and survey methodology, and the online survey included sections on respondent and organizational characteristics, current digital and data practices, AI awareness and experience, governance related to data responsibility, perceived barriers to AI adoption, and capacity-building needs.

#### 2.2.3 Language validation

The finalized English questionnaire was translated into Arabic. A bilingual public health professional then independently reviewed the Arabic version against the English original to ensure conceptual equivalence and consistency of terminology before dissemination.

The final instrument was pre-tested for clarity and functionality (e.g., item wording, skip logic, and completion time) prior to launch. Minor revisions were made based on feedback.

### 2.3 Participants and eligibility criteria

The target population comprised of professionals working in organizations operating in Qatar that contribute to humanitarian and development programs, coordination, preparedness, and/or international response support.

To support recruitment, the sampling frame was initiated through the project Technical Working Group (TWG), a multi-stakeholder group comprising 13 Qatar-based institutions and partners spanning government, UN/international agencies, academia, and humanitarian/development organizations.

#### 2.3.1 Inclusion criteria

Inclusion criteria were current employment in a humanitarian or development organization operating in or from Qatar (including organizations represented in the project TWG, spanning government, United Nations (UN)/international agencies, Non-Governmental Organizations (NGO)/charities, and academic partners), with a role directly involved in humanitarian/development programming or organizational strategy (including program design and management, field operations, information management, monitoring and evaluation, partnerships, or executive leadership). Participants were required to be 18 years or older and willing and able to provide electronic informed consent.

These roles were selected because they involve direct engagement with program implementation, organizational data, operational decision-making, monitoring, partnerships, or strategic planning. Professionals in these functions were therefore expected to have sufficient familiarity with their organizations’ digital practices, workforce capabilities, operational constraints, and governance arrangements to provide informed assessments of AI readiness. Prior technical expertise in AI was not required because the study also aimed to capture variation in AI awareness, experience, and perceived preparedness across the sector.

#### 2.3.2 Exclusion criteria

Exclusion criteria were roles that were exclusively administrative and not directly related to program data or strategy (e.g., general human resources or facility management), volunteers or interns not involved in strategic or programmatic functions, and individuals employed by organizations outside the humanitarian and development sector.

### 2.4 Recruitment and data collection

A purposive and snowball sampling strategy was employed, initiated through the established Technical Working Group (TWG). This TWG comprises representatives from 13 institutions, reflecting key humanitarian and development actors in Qatar. The survey link and a formal invitation were first shared with members of the TWG, then they were asked to disseminate the survey link to eligible colleagues within their respective organizations and to their broader professional networks. This snowballing approach was chosen to maximize reach across the diverse and interconnected humanitarian community. As a result, the sample should be interpreted as a stakeholder-based snapshot rather than a representative sample of all humanitarian and development actors in Qatar. The Ministry of Foreign Affairs and QFFD as Chair and Co-Chair of the TWG have supported the dissemination of the survey across the TWG members and their organizations.

Data collection was conducted entirely online via the Qatar University Blue survey platform between November 25, 2025, and January 30, 2026 (a period immediately preceding analysis). Because the survey link was disseminated through professional networks via forwarding, the total number of individuals who received the invitation could not be determined; therefore, a formal response rate could not be calculated. As a result, findings should be interpreted as descriptive of participating respondents, and the potential for self-selection bias should be considered when interpreting levels of readiness and perceived barriers.

### 2.5 Framework operationalization and measures

For reporting and interpretation, survey items were organized using the Organizational Digital Transformation Readiness model and the Unified Theory of Acceptance and Use of Technology as analytical lenses. ODTR domains were used to structure organizational readiness conditions (technological resources, management capability/governance, human capability, and organizational culture), while UTAUT-aligned constructs were used to organize adoption-related perceptions (performance expectancy, effort expectancy, social influence, and facilitating conditions).

UTAUT and ODTR were selected because they capture complementary levels of readiness relevant to humanitarian AI implementation. Compared with technology-acceptance models focused mainly on perceived usefulness and ease of use, UTAUT additionally considers social influence and facilitating conditions, which are important in humanitarian organizations where technology use is shaped by professional norms, institutional mandates, and access to organizational support. ODTR was selected over more general organizational-change models because it explicitly assesses digital-transformation conditions across technological resources, management capability, human capability, and organizational culture. These domains are particularly relevant in humanitarian settings, where AI adoption depends not only on staff willingness but also on data responsibility, governance, safeguards, technical infrastructure, and institutional capacity. Integrating the two frameworks therefore allowed the study to examine whether favorable individual adoption perceptions were matched by the organizational conditions required for responsible implementation.

**Figure 1:**
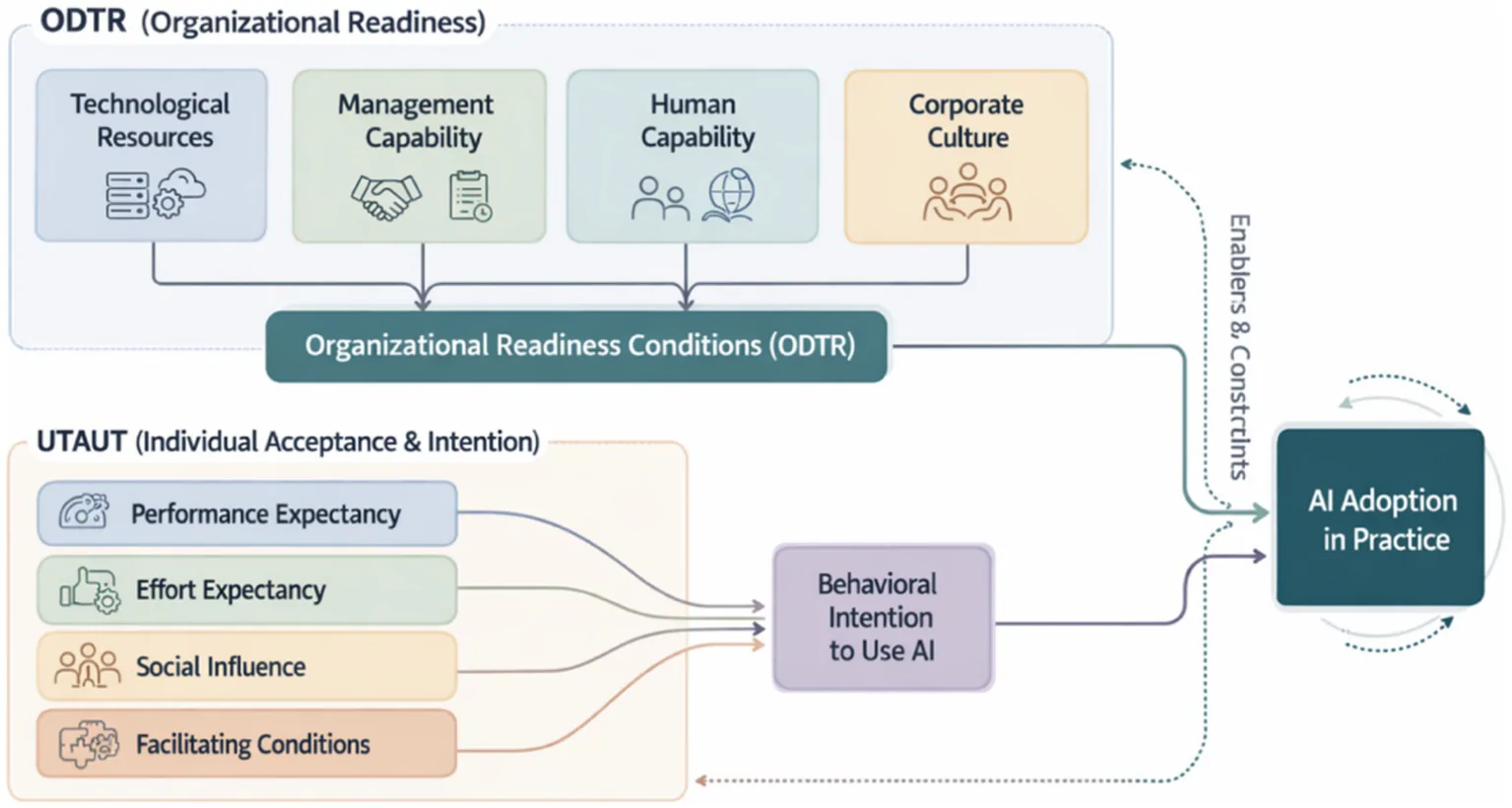
Integrated ODTR-UTAUT conceptual framework used in this study.

The purpose of combining these frameworks was not to test a causal model, but to enable structured comparison between individual adoption-related perceptions and organizational readiness conditions, thereby supporting interpretation of whether readiness appeared aligned or unevenly distributed across levels. The integrated framework used in this study is shown in Figure 1. Item development and construct assignment followed a theory-informed, deductive process. Survey items were developed from the conceptual definitions of the ODTR domains and UTAUT constructs, together with relevant humanitarian guidance on responsible data and AI. Each item was assigned to the single domain or construct that most closely reflected its primary conceptual focus. Item wording and contextual relevance were subsequently examined through Technical Working Group consultation, external expert review, bilingual language review, and pretesting. These procedures supported face validity, content relevance, and conceptual alignment within the humanitarian context; however, they did not constitute formal psychometric validation. The resulting construct assignments were therefore used for structured descriptive reporting rather than confirmatory testing of the frameworks.

### 2.6 Outcomes and operational definitions

The survey assessed three primary outcomes: (1) Individual AI readiness, measured with the binary item, “Are you ready to use AI in your work?” (yes/no); (2) Organizational AI adoption outlook, measured with the item, “Does your organization intend to use more AI in the coming years?” (Yes/No/I do not know); and (3) Organizational AI readiness conditions, assessed through grouped indicators covering governance, perceived barriers, and capacity-building needs. These outcomes were assessed using single items to minimize respondent burden and provide direct descriptive indicators within an exploratory survey designed for busy humanitarian professionals. They were not intended to function as multi-item psychometric scales or to measure latent constructs comprehensively. Accordingly, internal-consistency reliability cannot be estimated for these individual outcomes, and the results should be interpreted as descriptive indicators of respondents’ reported readiness and organizational outlook.

### 2.7 Data management and analysis

Survey responses were exported from the collection platform into Excel for cleaning and descriptive analysis. Frequencies and percentages were used to summarize categorical variables. Capacity-building priorities were identified by calculating the gap between mean perceived skill importance and mean self-rated competence. Thematic coding was used to analyze open-ended responses.

Survey items were assigned to ODTR domains and UTAUT-informed constructs to support structured reporting. Composite ODTR and UTAUT index scores were calculated by rescaling item responses to a 0–1 range and averaging within domains and overall; scores were multiplied by 100 for reporting. Multi-select items were excluded from index scoring, and “I do not know/not applicable” responses were treated as missing for index calculations.

Internal-consistency statistics were not calculated for the composite indices because the indices were developed as formative descriptive summaries of conceptually related but heterogeneous readiness indicators rather than as reflective psychometric scales. The included indicators represented distinct aspects of readiness and were not assumed to be interchangeable manifestations of a single latent construct. Consequently, a high degree of inter-item correlation was neither required nor necessarily expected, and statistics such as Cronbach’s alpha could provide a misleading assessment of index quality. The composite scores should therefore be interpreted as exploratory descriptive indices, and future research should formally evaluate their reliability and construct validity using larger samples and dedicated scale-validation procedures.

For the ODTR human capability domain, composite readiness scores were calculated using items that reflect current capability (self-rated competence and related skill indicators). Importance ratings were analyzed separately as indicators of training need (importance − competence) and were not included in readiness scoring to avoid conflating need with capability.

### 2.8 Ethics

The study received ethical approval from the Qatar University Institutional Review Board (QU-IRB #ID 23289895-1). The study was conducted in accordance with the Declaration of Helsinki. All participants were provided with a digital information sheet outlining the study’s purpose and confirming the voluntary and confidential nature of their participation. Electronic informed consent was obtained from all respondents before they began the survey. Survey responses were collected anonymously through the Qatar University Blue survey platform and did not include direct personal identifiers. Following export for analysis, the de-identified dataset was stored in a password-protected Qatar University institutional OneDrive folder, with access restricted to authorized members of the research team. The data will be retained and securely disposed of in accordance with the approved IRB protocol and Qatar University’s institutional data-retention requirements.

## 3 Results

### 3.1 Participant and organizational characteristics

Table 1 represents the sample profile (*N* = 83). The median age was 41 years (IQR 34.5–48; range 25–70). 65.1% of the respondents were male, and the highest completed degree was a master’s degree for (54.2%). Participants were affiliated with UN/international organizations (42.2%), governmental organizations (25.3%), and local NGOs (24.1%). Sector representation included health (32.5%) and other sectors (39.8%) including education, logistics/supply chain, protection, community engagement, and cross-cutting coordination/support functions. Organizational tenure was >7 years for 35 participants (42.2%). Job functions (multiple selections allowed) included program/field operations (33.7%) and executive/director roles (28.9%) among others. The sample included respondents from governmental organizations, United Nations and international organizations, local nongovernmental organizations, and multiple professional and sectoral backgrounds. However, no comprehensive census or sampling frame describing the full composition of Qatar’s humanitarian and development workforce was available for comparison. It was therefore not possible to determine whether the distribution of organization types, sectors, or professional roles in the sample was statistically representative of the wider target population.

**Table 1:** Characteristics of participants.

Characteristic	Category	*N* (%) = 83
Gender	Male	54 (65.1%)
	Female	29 (34.9%)
Highest degree completed	Bachelor’s	29 (34.9%)
	Master’s	45 (54.2%)
	PhD/MD	7 (8.4%)
	Other	2 (2.4%)
Organization type	United Nations/international organization	35 (42.2%)
	Governmental organization	21 (25.3%)
	Local NGO	20 (24.1%)
	University/academic	4 (4.8%)
	Other	3 (3.6%)
Sector	Health	27 (32.5%)
	Education	9 (10.8%)
	WASH	13 (15.7%)
	Protection	1 (1.2%)
	Other	33 (39.8%)
Years with current organization	< 1 year	12 (14.5%)
	1–3 years	21 (25.3%)
	4–6 years	15 (18.1%)
	> 7 years	35 (42.2%)
Job title/function (multi-select)	Program/field operations	28 (33.7%)
	Executive/director	24 (28.9%)
	Monitoring & evaluation/research	10 (12.0%)
	Data/information & data management	4 (4.8%)
	Finance/admin	3 (3.6%)
	Other	14 (16.9%)

### 3.2 UTAUT and readiness

This subsection reports UTAUT-informed indicators of AI adoption, including proxy measures for performance expectancy and social influence, alongside readiness-related outcomes.

#### 3.2.1 Perceived organizational AI adoption outlook and individual readiness

**Figure 2:**
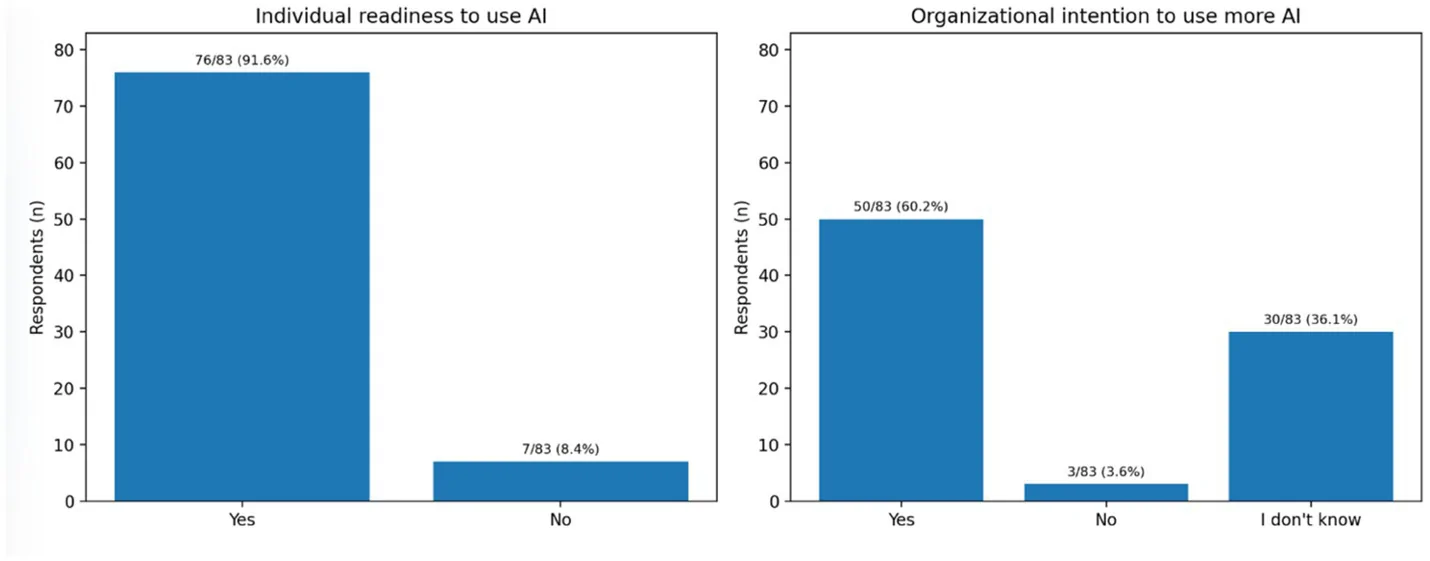
Perceived organizational intention and individual readiness to use AI (*N* = 83).

In total, 60.2% of respondents reported that their organization intends to use more AI in the coming years, while 30 indicated they do not know and 3 reported no intention. At the individual level, 91.6% reported being ready to use AI, while 7 reported not being ready (Figure 2).

#### 3.2.2 Performance expectancy: perceived operational areas where AI could add value

**Figure 3:**
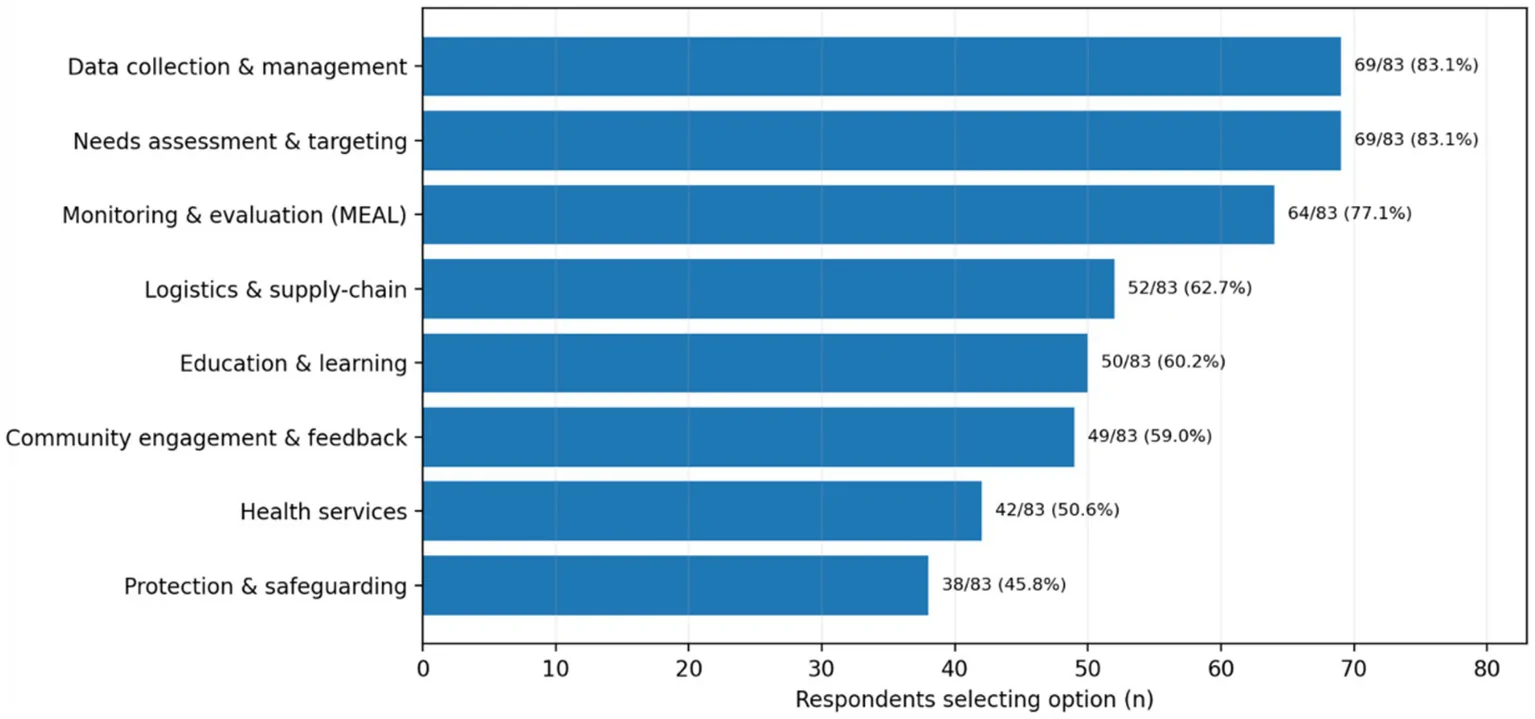
Perceived operational areas where AI could add value (multi-select; *N* = 83).

Respondents identified potential value for data collection and management (83.1%) and needs assessment and targeting of assistance (83.1%), followed by monitoring and evaluation (77.1%). Additional areas included logistics and supply-chain (62.7%), education and learning (60.2%), and community engagement and feedback (59.0%). Approximately half identified benefits for health services (50.6%), while others selected protection and safeguarding (45.8%). A small proportion selected “other” (3.6%) without providing additional detail (Figure 3).

Open-ended responses describing current AI use were consistent with these perceived value areas. Respondents most frequently cited general-purpose generative AI assistants (e.g., ChatGPT/Copilot) and uses related to data collection/management, monitoring and reporting, and needs assessment/targeting.

#### 3.2.3 Perceived organizational openness to AI (UTAUT social influence proxy)

This item was treated as a proxy for UTAUT social influence, reflecting respondents’ perceptions of organizational norms and support for AI use. 81.9% of the respondents agreed that their organization is open to using AI in its work (68/83), while 11 (13.3%) reported not knowing/not applicable and 4 (4.8%) disagreed.

### 3.3 ODTR and readiness

#### 3.3.1 Digital and data foundations

Respondents reported frequent engagement with digital data tools and generally high comfort working with data. Nearly two-thirds indicated that their work always involves entering, analyzing, or reviewing data using digital tools (63.9%), while 34.9% reported doing so sometimes and 1 respondent reported never. Most respondents agreed that they are comfortable using digital data (such as Excel, Kobo, DHIS2, and other data-entry or analysis platforms) in their work (73, 88.0%), whereas 7 (8.4%) disagreed and 3 (3.6%) reported that this was not applicable.

**Figure 4:**
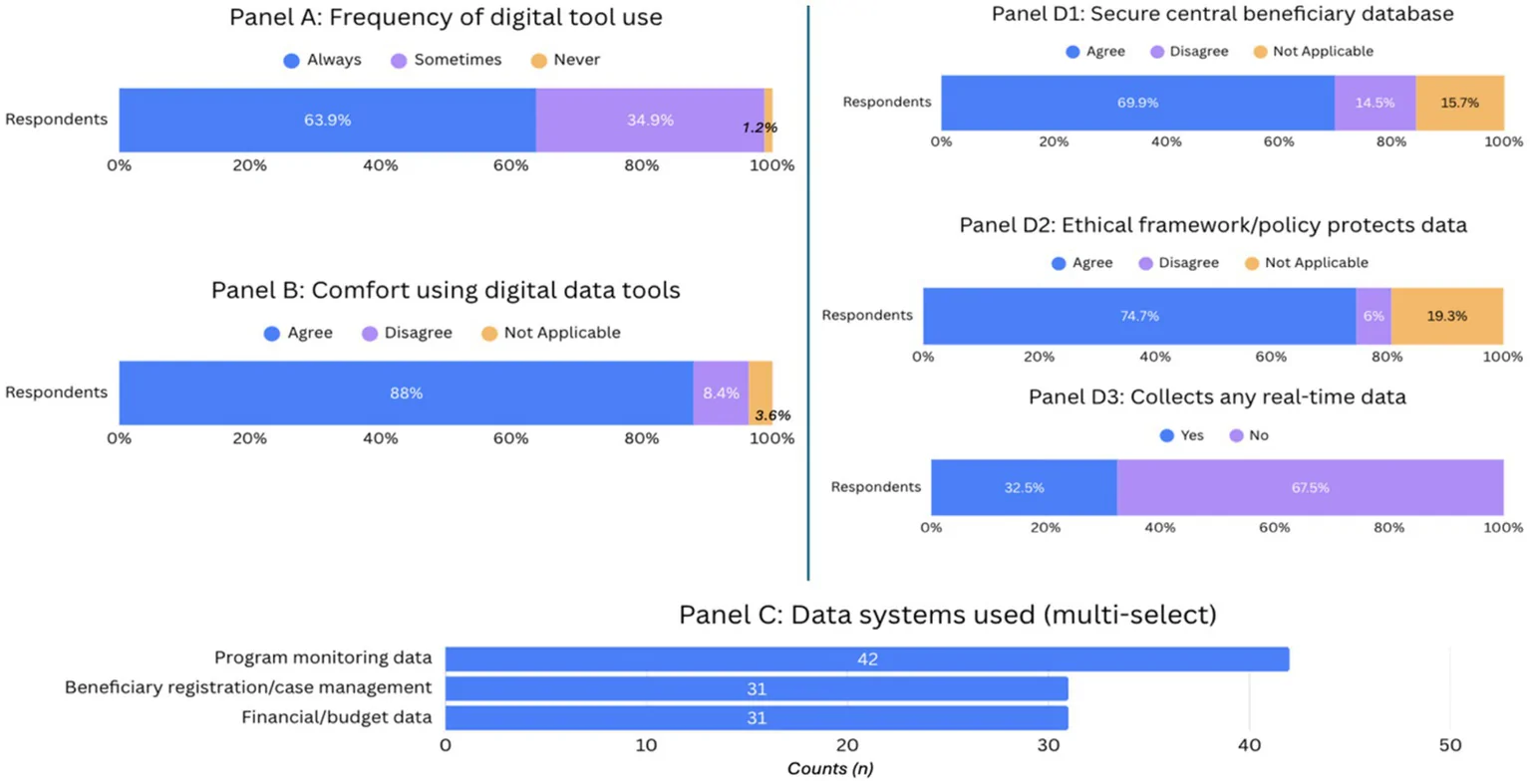
Digital and data foundations relevant to responsible AI adoption (*N* = 83).

Because many AI applications depend on reliable, well-governed organizational data, existing data systems and data protection policies are foundational readiness conditions for responsible AI adoption. The most reported data systems used within organizations included program monitoring data (42, 50.6%), beneficiary registration or case management systems (31/83, 37.3%), and financial/budget data (31, 37.3%) (multiple selections allowed). In addition, 58, (69.9%) agreed that beneficiary data are stored in a secure, central database, while 12 (14.5%) disagreed and 13 (15.7%) reported not knowing. Respondents also reported that their organization has an ethical framework or policy that protects data (62, 74.7%), while 5/83 (6.0%) reported no such policy and 16 (19.3%) reported not knowing. By contrast, real-time data collection (i.e., data collected and available with minimal delay, such as mobile reporting, social media monitoring, sensors, or rapid feedback mechanisms) was less common: 27 (32.5%) reported collecting any real-time data, whereas 56 (67.5%) reported not doing so (Figure 4).

#### 3.3.2 Governance and safeguards

Governance and oversight mechanisms relevant to responsible AI adoption were variably reported. 44 respondents agreed that their organization has written Standard Operating Procedures (SOPs) covering privacy and fair data use (53.0%), while 12 (14.5%) disagreed and 27 (32.5%) reported not knowing or that this was not applicable.

Formal data-sharing arrangements with external partners were less consistently reported: 21 (25.3%) reported having a data-sharing Memorandum of Understanding (MoU), 25 (30.1%) reported no MoU, and 37 (44.6%) reported not knowing. Independent ethics review for digital or AI-related projects was reported by 15 (18.1%), while 28 (33.7%) reported no such review process and 40 (48.2%) reported not knowing. Similarly, 23 (27.7%) reported that their organization has developed or adapted an AI ethics framework or principles (or is planning to do so), whereas 13 (15.7%) reported no framework and 56.6% reported not knowing.

### 3.4 Perceived barriers to AI adoption

**Figure 5:**
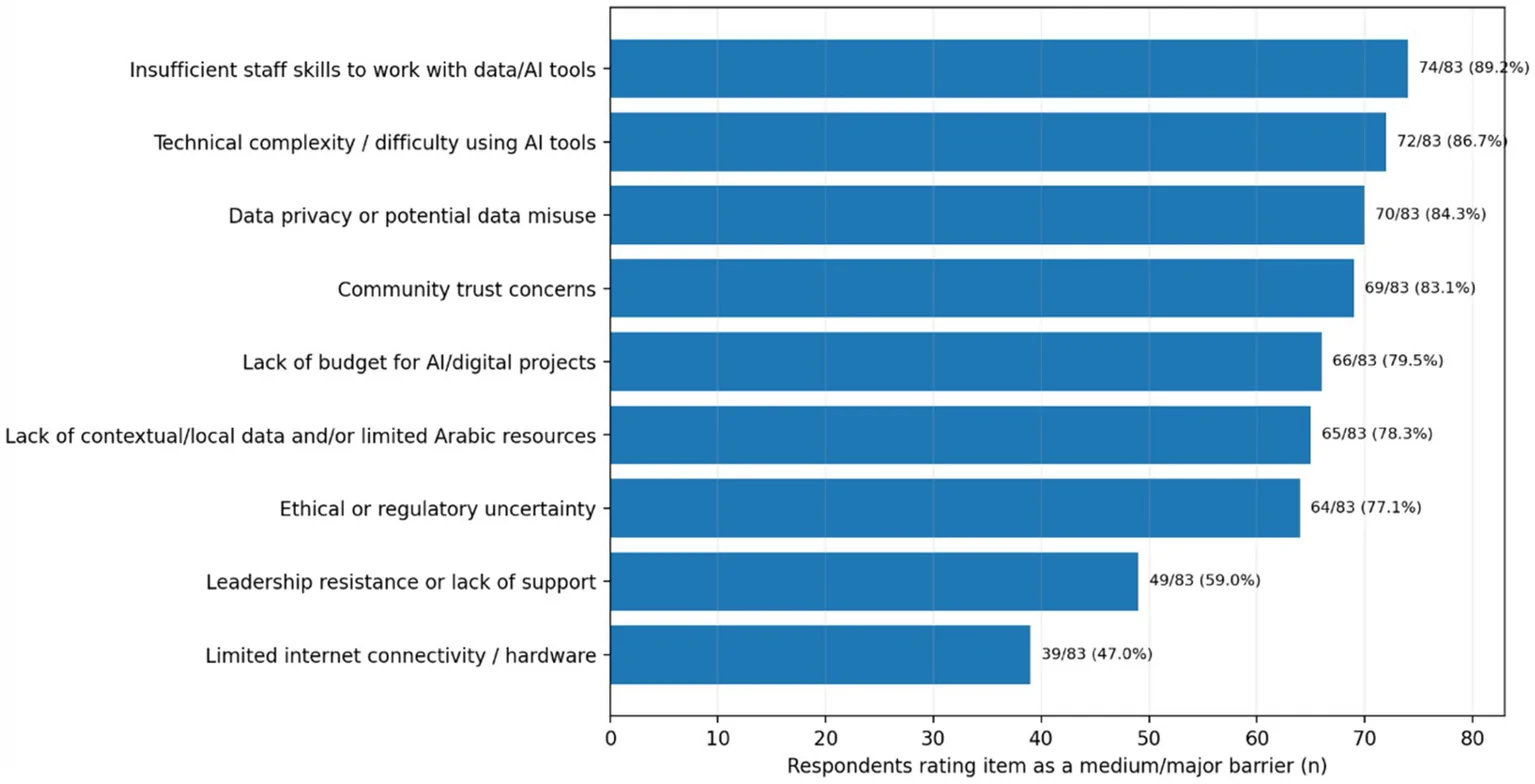
Perceived barriers to AI adoption (*N* = 83).

Respondents most frequently reported capacity- and complexity-related constraints (Figure 5). 74 of the participants reported that a medium/ major barrier was insufficient staff skills to work with data or AI tools (89.2%; major: 36.1%) and technical complexity or difficulty of using AI tools (86.7%; major: 30.1%). High levels of concern were also reported for data privacy or potential data misuse (84.3%; major: 39.8%) and community trust concerns (83.1%; major: 22.9%). Resource constraints were also a concern with lack of budget for AI or digital projects reported as a medium/major barrier by 66/83 (79.5%). Other reported barriers included lack of contextual/local data and/or limited Arabic-language resources (78.3%), and ethical or regulatory uncertainty (77.1%). Leadership resistance or lack of support was less frequently cited (59.0%), while limited internet connectivity or hardware was the least commonly reported barrier (47.0%).

Open-ended responses provided additional context, frequently emphasizing the importance of clear guidance on data protection and safe use, practical staff training, and access to relevant data resources to support implementation.

### 3.5 Capacity-building needs: importance vs. competence

**Figure 6:**
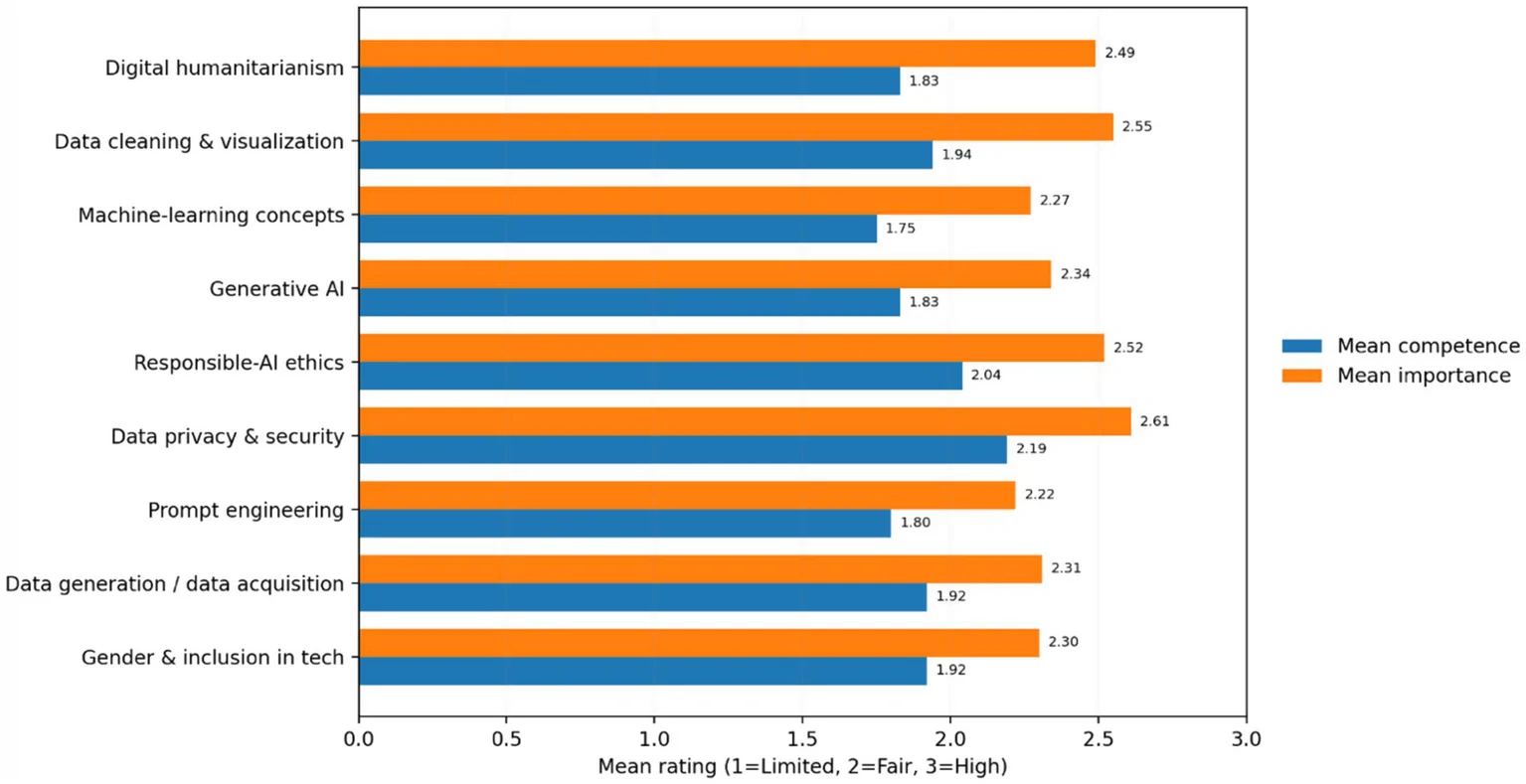
Perceived training needs: Importance vs. competence across domains (*N* = 83).

Across nine training domains, respondents consistently rated these skills as important for their work, while self-rated competence was lower, indicating a broad perceived need for capacity-building (Figure 6). Table 2 summarizes capacity-building priorities using the mean gap between perceived importance and self-rated competence (importance − competence; scale 1 = Limited, 2 = Fair, 3 = High).

**Table 2:** Training priority gaps between perceived importance and self-rated competence across domains (*N* = 83).

Training domain	Mean competence	Mean importance	Gap (importance − competence)
Digital humanitarianism	1.83	2.49	0.66
Data cleaning & visualization	1.94	2.55	0.61
Machine-learning concepts	1.75	2.27	0.52
Generative AI	1.83	2.34	0.51
Responsible-AI ethics	2.04	2.52	0.48
Data privacy & security	2.19	2.61	0.42
Prompt engineering	1.80	2.22	0.42
Data generation / data acquisition	1.92	2.31	0.39
Gender & inclusion in tech	1.92	2.30	0.38

The largest gaps were observed for digital humanitarianism (gap 0.66) and data cleaning and visualization (gap 0.61), followed by machine-learning concepts (gap 0.52) and generative AI (gap 0.51). Gaps were also notable for responsible-AI ethics (gap 0.48) and for data privacy and security and prompt engineering (gap 0.42 each), indicating that perceived training needs exceeded current self-rated competence across all domains. Open-ended comments highlighted interest in practical training focused on using AI for routine workflows while maintaining verification and responsible data practices.

### 3.6 Composite ODTR and UTAUT index scores (0–100)

Composite ODTR and UTAUT-informed index scores were examined to characterize the overall readiness pattern in this stakeholder sample and to compare adoption-related perceptions with organizational readiness conditions. Indices were computed by rescaling item responses to a 0–1 range and averaging within domains and overall (multi-select items were excluded from scoring; ‘I do not know/not applicable’ responses were treated as missing) (see Table 3).

**Table 3:** ODTR and UTAUT index scores (overall; *N* = 83).

Index/domain	Mean	SD	Median	IQR	Min	Max
ODTR – Technological resources	55.4	34.9	50.0	50.0	0.0	100.0
ODTR – Management capability (governance)	43.6	18.4	45.0	23.1	0.0	100.0
ODTR – Human capability (perceived competence)	55.5	23.5	58.3	26.5	4.2	100.0
ODTR – Corporate culture	75.7	35.1	100.0	50.0	0.0	100.0
ODTR – Overall index	**56.8**	**19.1**	**60.0**	**22.5**	**8.5**	**96.4**
UTAUT – Social influence	94.4	23.1	100.0	0.0	0.0	100.0
UTAUT – Effort expectancy	89.2	31.3	100.0	0.0	0.0	100.0
UTAUT – Behavioral intention	91.6	28.0	100.0	0.0	0.0	100.0
UTAUT – Overall index	**90.8**	**19.3**	**100.0**	**0.0**	**0.0**	**100.0**

Bold values indicate the overall ODTR and UTAUT index scores.

Overall, the UTAUT-informed index was high (mean 90.8, SD 19.3; median 100.0), reflecting favorable individual-level adoption-related perceptions captured by the proxy indicators used in this study (organizational openness to AI, perceived ease of learning/using AI tools, and self-reported readiness). The median of 100.0 and IQR of 0.0 across UTAUT components indicates that at least half of respondents selected the highest response category on these binary proxy items, while the observed SD and minimum values indicate some variability across respondents.

By contrast, the ODTR overall index was moderate (mean 56.8, SD 19.1; median 60.0), indicating more mixed readiness conditions across organizational domains. Among ODTR domains, management capability/governance had the lowest mean score (mean 43.6, SD 18.4), suggesting comparatively lower visibility or consistency of governance and safeguard mechanisms. Technological resources and human capability (competence) were moderate on average (means 55.4 and 55.5, respectively), with wide ranges indicating variability across respondents and organizations. Corporate culture scored higher on average (mean 75.7, SD 35.1; median 100.0), indicating that respondents commonly perceived an enabling climate for innovation even when other readiness conditions were less consistently in place. Overall, UTAUT-informed scores were higher than ODTR scores in this sample, and governance was the lowest-scoring ODTR domain.

Taken together, these scores suggest an uneven readiness configuration in which respondents reported favorable conditions for interest and openness toward AI, but less consistent organizational readiness to govern and support its use.

The scoring approach should be considered when interpreting these indices. Rescaling and averaging placed all included indicators on a common 0–100 metric and gave them equal weight, enabling within-sample comparison across readiness domains; however, the resulting values do not represent the percentage of an organization that is “ready” and should not be interpreted using validated readiness thresholds. Treating “I do not know/not applicable” responses as missing means that the indices summarize available substantive responses but may not fully capture uncertainty, which may itself indicate limited governance visibility. In addition, the use of binary proxy indicators in the UTAUT-informed index contributed to the concentration of scores near the upper end of the scale. Direct numerical comparison with previous readiness studies is limited because studies have used different frameworks, items, weighting procedures, populations, and implementation contexts. Nevertheless, the observed contrast between favorable individual adoption perceptions and more variable organizational conditions is qualitatively consistent with literature describing AI readiness as multidimensional and dependent on institutional capacity, leadership, governance, and implementation structures (35, 36).

## 4 Discussion

This study provides an exploratory view of AI readiness as perceived by a stakeholder sample within Qatar’s humanitarian and development ecosystem. The main analytical insight is that readiness did not appear as a single, uniform condition. Instead, the findings point to an uneven readiness configuration in which adoption-related perceptions at the individual level were markedly more favorable than organizational readiness conditions, particularly those related to governance and safeguards. Interpreted through the combined ODTR-UTAUT lens, this pattern suggests that interest in and openness to AI may emerge earlier than the institutional routines, oversight mechanisms, and capability foundations required for its consistent and responsible use.

### 4.1 A layered readiness pattern across individual and organizational levels

Respondents commonly expressed openness to AI, perceived it as useful for a range of operational tasks, and reported being personally ready to use it in their work. At the same time, organizational conditions related to governance, safeguards, and capability were reported less consistently. Interpreted through the combined lenses of the Unified Theory of Acceptance and Use of Technology and Organizational Digital Transformation Readiness, this pattern suggests that enthusiasm for AI use may emerge earlier than the institutional structures needed to support its responsible and consistent implementation (30, 31).

This distinction is important because it avoids reducing readiness to a single score or binary state. UTAUT directs attention to adoption-related perceptions such as perceived usefulness, ease of use, and normative support, while ODTR focuses on organizational enablers such as governance, technological resources, human capability, and culture (35). Considering these dimensions together allows readiness to be understood as unevenly distributed across levels rather than uniformly present or absent. In this sample, respondents appeared more likely to report favorable conditions for willingness and openness than for formal organizational preparedness (36). This interpretation is consistent with broader discussions in the organizational AI adoption literature, where readiness has been described as multidimensional and dependent not only on technical access, but also on institutional capacity, leadership, and implementation structures (35, 36).

However, the relationship between individual acceptance and organizational readiness may not follow the same sequence in every setting. Technology-adoption and organizational-readiness research also positions facilitating conditions, leadership support, and institutional capability as antecedents that can shape individual willingness and sustained technology use (30, 31, 35, 36). Moreover, the adoption of formal AI policies by some humanitarian institutions demonstrates that governance arrangements may be developed proactively, before or alongside widespread operational uptake, rather than only after individual experimentation has become established (19). These perspectives suggest that individual and organizational readiness may develop sequentially, concurrently, or through reciprocal influence depending on institutional maturity, leadership priorities, and regulatory context. The layered pattern observed in the present sample should therefore be interpreted as context-specific rather than as a universal pathway of humanitarian AI adoption.

The findings also suggest that organizational culture and governance may not develop in parallel. In the present sample, corporate culture scores were relatively high, indicating that respondents often perceived their organizations as open to innovation and new technologies. However, governance-related indicators were weaker and less consistently reported, particularly with respect to AI ethics frameworks, data-sharing agreements, and ethics review mechanisms (37). This combination may indicate that an enabling climate for experimentation can coexist with limited visibility of formal oversight arrangements (37). Rather than concluding that governance is absent, a more cautious interpretation is that governance mechanisms may be unevenly operationalized, inconsistently communicated, or still emerging within participating organizations. This is an important distinction in early-stage AI adoption, where organizational interest may outpace the development of clear institutional routines and safeguards (37).

This layered readiness pattern has practical significance for humanitarian and development settings, where the consequences of poorly governed technology use may be especially serious. In such contexts, individual experimentation with AI tools may create immediate efficiencies, but it may also introduce uncertainty around privacy, accountability, verification, and appropriate use if organizations have not yet established clear guidance and supporting processes. Humanitarian scholarship has repeatedly emphasized that technological innovation in high-stakes settings must be assessed not only in terms of utility, but also in relation to duty of care, data responsibility, and the potential for unintended harm (18, 19, 23). Viewed in this light, the contrast observed in this study between favorable individual readiness and more mixed organizational conditions is not simply a gap; it is a useful signal of where implementation support may be most needed.

### 4.2 The governance imperative in a high-stakes sector

The findings suggest that governance-related readiness remains less visible and less consistently reported than other dimensions of AI readiness within this stakeholder sample. Among the ODTR domains, management capability and governance had the lowest composite score, and respondents were less certain about the presence of formal mechanisms such as AI ethics frameworks, data-sharing agreements, and ethics review processes. This does not necessarily indicate that governance is absent across participating organizations; rather, it suggests uneven visibility, inconsistent operationalization, or limited respondent awareness of the mechanisms that do exist. In exploratory survey-based research of this kind, that distinction is important, because perceived readiness reflects not only institutional arrangements themselves but also how clearly they are communicated, understood, and embedded in routine practice.

The survey directly supports conclusions only about respondents’ reported awareness and perceptions of governance mechanisms; it did not independently verify organizational policies, assess their implementation quality, or measure resulting harms or operational outcomes. The broader implications discussed below are therefore conceptual interpretations informed by humanitarian and responsible-AI literature rather than effects demonstrated by the present data.

This pattern is analytically important in humanitarian and development settings, where AI use intersects with sensitive data, high-stakes decisions, and obligations related to accountability and protection (20). In such contexts, governance is not only a matter of formal policy existence, but of whether practical safeguards are in place to shape how tools are selected, used, monitored, and reviewed (38). Existing humanitarian and responsible AI guidance has consistently emphasized the importance of clear roles, risk assessment, proportionality, and harm mitigation when deploying data-driven technologies (19, 23, 37). The comparatively weaker governance profile observed in this study therefore matters not because it proves institutional failure, but because it may signal that important enabling conditions for responsible AI use are still emerging or are not yet sufficiently visible to staff.

The survey findings on barriers reinforce this interpretation. Concerns related to data privacy, potential misuse, and community trust were among the most frequently reported barriers, suggesting that respondents view governance and responsible use as central implementation issues rather than secondary considerations. This aligns with broader humanitarian literature arguing that the risks associated with AI adoption are not only technical, but social, ethical, and institutional, particularly where affected populations may have limited ability to challenge, understand, or opt out of data-driven decisions (18). Humanitarian innovation cannot be separated from questions of accountability, transparency, and potential harm (12, 39). In this sense, the governance-related findings in the present study should be interpreted less as evidence of “low maturity” in an absolute sense and more as an indication that governance remains a salient and possibly unsettled area of organizational readiness in this context.

From an implementation perspective, both possibilities matter. If safeguards are not in place, organizations may face increased risks in adopting AI tools (40). If safeguards do exist but are not widely known, then internal communication, training, and operational integration may be insufficient to support consistent use in practice (41).

Taken together, these findings suggest that strengthening AI readiness in humanitarian and development settings may require more than technical access or general openness to innovation. It may also require translating broad ethical commitments into practical and visible organizational arrangements that staff can recognize and apply in day-to-day work. This includes not only formal policies, but also usable procedures, defined oversight responsibilities, risk screening processes, and clear guidance on appropriate use. In early-stage settings such as the one examined here, improving the visibility and operationalization of such safeguards may be an important step toward aligning organizational preparedness with growing staff interest in AI.

### 4.3 Pragmatic ambitions, foundational gaps, and strategic funding

The findings suggest that respondents most often associated AI with practical applications that could support immediate improvements in routine work. Data collection and management, needs assessment and targeting, and monitoring and evaluation were among the most frequently identified areas where AI could add value. This pattern indicates that, within this stakeholder sample, AI was viewed less as a transformative or autonomous system and more as a tool for strengthening existing workflows and improving efficiency in information-intensive tasks. Such a pragmatic orientation is consistent with wider discussions of early AI adoption in organizations, where initial uptake often centers on augmenting established functions before moving toward more complex or automated uses (42, 43). These priorities are also relevant in humanitarian and disaster-related contexts, where timely information processing, monitoring, and operational planning are central to coordination and decision-making (44).

These pragmatic ambitions may nevertheless be constrained by foundational data and AI competencies, consistent with evidence that effective AI adoption depends on underlying skills rather than software access alone (45, 46). Based on their reported frequency and proximity to implementation, three priority areas emerge. First, insufficient staff skills (89.2%) and technical complexity (86.7%) represent immediate operational constraints because they may limit staff members’ ability to select, use, verify, and monitor AI tools effectively. Second, concerns about data privacy or potential misuse (84.3%) and community trust (83.1%) represent governance and legitimacy risks that are particularly consequential in humanitarian settings involving sensitive data and vulnerable populations. Third, insufficient budget for AI or digital projects (79.5%) may restrict organizations’ ability to invest in secure infrastructure, specialized expertise, training, and oversight. Although these descriptive frequencies do not establish which barriers have a causal effect on implementation, they suggest that workforce capability, usable technical support, and responsible governance should be treated as immediate and interconnected priorities rather than addressed in isolation.

Viewed in this way, the findings support a more sequenced understanding of capacity-building. In early-stage settings, strengthening readiness may require starting with core competencies such as data literacy, data handling, verification practices, and responsible data use before expecting widespread uptake of more advanced AI applications. This does not imply that organizations must fully complete one stage before beginning another, but it does suggest that technical experimentation without adequate foundational capability may limit effective and trusted use. Similar arguments have been made in the organizational readiness literature, where AI preparedness is understood as a capacity and governance issue as much as a technological one (35, 36).

Strategic support should extend beyond acquiring AI tools or funding isolated short-term pilots. Concrete interventions could include role-specific training in data literacy, output verification, privacy, and responsible AI; designated internal AI focal points; and approved-use guidance specifying which tools and data may be used for particular tasks. Cross-functional governance groups could oversee risk screening, documentation, human review of consequential outputs, incident reporting, and periodic monitoring. Funders and coordinating bodies could support smaller organizations through shared training resources, model policies, secure testing environments, and pilot funding linked to governance and evaluation requirements. Resources developed by NetHope and the Humanitarian Leadership Academy illustrate how these principles can be translated into practical guidance (22, 47).

Importantly, the present findings do not justify broad prescriptions about what all humanitarian organizations should fund or prioritize. Rather, they point to a context-specific pattern within this sample: respondents identified practical use cases for AI, but also reported foundational gaps that may hinder implementation. In this sense, the results suggest that strategic support for AI readiness may be most effective when it is aligned not only with technological ambition, but also with the more basic organizational capacities needed to use such tools safely, confidently, and consistently.

### 4.4 Strengths and limitations

This study offers an exploratory empirical snapshot of AI readiness among stakeholders working within Qatar’s humanitarian and development ecosystem. A key strength is its use of a structured dual-lens approach, using ODTR and UTAUT to distinguish between organizational readiness conditions and individual adoption-related perceptions. This allowed the study to move beyond a simple inventory of attitudes by examining how readiness appeared to be distributed across different levels and domains within the participating sample. The bilingual survey format also supported inclusion across a diverse professional environment, while the incorporation of open-ended responses provided additional contextual insight into how respondents understood current and potential AI use in practice.

At the same time, several limitations should be considered when interpreting the findings. First, the study was based on a relatively small stakeholder sample and used purposive and snowball recruitment initiated through an existing Technical Working Group network. As a result, the findings should be interpreted as a descriptive, stakeholder-based snapshot rather than a representative assessment of all humanitarian and development actors operating in or from Qatar. The sampling approach may also have introduced self-selection bias, including possible overrepresentation of respondents with stronger interest in digital innovation or AI-related topics. Recruitment through established professional networks may also have favored larger or more institutionally connected organizations while underrepresenting smaller organizations and professionals outside these networks, potentially reducing sample diversity and further limiting the generalizability of the findings. The modest sample size, unequal numbers across organization types, and use of overlapping multiple-selection categories for professional roles also limited the reliability of stratified comparisons; such analyses could produce unstable estimates and increase the likelihood of chance findings.

Second, the study relied primarily on self-reported perceptions. This reliance on data collected through a single self-report survey may have introduced recall bias, social-desirability bias, and common-method bias, potentially inflating observed consistency among related perceptions or encouraging respondents to report more favorable attitudes toward AI. Accordingly, the findings reflect respondents’ reported experiences, views, and awareness rather than independently verified organizational practices or formal institutional capacity. This is particularly important for governance-related items, where high proportions of “do not know” responses may reflect either the absence of formal mechanisms or limited visibility of arrangements that do exist. The study therefore cannot determine the actual maturity or effectiveness of organizational governance systems and should not be interpreted as an audit of institutional readiness.

Third, although survey items were organized using ODTR and UTAUT-informed constructs, the study did not apply UTAUT as a full validated psychometric instrument or test a causal explanatory model. In addition, several primary outcomes and framework-aligned constructs were represented by single-item indicators, which may not capture the full conceptual breadth of the constructs and may reduce measurement reliability. Rather, selected items were used as conceptually aligned proxy indicators to support structured descriptive interpretation. Similarly, the composite scores reported in this study should be understood as summary descriptive indices designed to characterize patterns within the sample, not as validated diagnostic scales. While this approach was appropriate for an exploratory baseline assessment, it limits the strength of conclusions that can be drawn regarding construct validity, causal relationships, or differences between organizations.

Finally, the cross-sectional design captures perceptions at a single point in time within a rapidly evolving technological environment. Consequently, the study cannot establish causal relationships among the readiness factors examined or determine how individual and organizational readiness changes over time. Organizational policies, staff familiarity with AI, and implementation practices may shift quickly, particularly as generative AI tools become more accessible and more formally discussed across institutions. Follow-up studies using larger and more systematic samples, longitudinal designs, and qualitative organizational case studies would help deepen understanding of how readiness develops over time and how governance and practice evolve in relation to growing interest in AI.

### 4.5 Future research

A primary methodological priority is the formal validation of the integrated ODTR-UTAUT readiness framework. Larger studies should develop multi-item measures for each construct and assess internal consistency, factor structure, convergent and discriminant validity, and sensitivity to changes in readiness over time. Once validated, a harmonized instrument could support multicountry comparative studies across humanitarian and development ecosystems with different governance structures, institutional mandates, resource levels, and degrees of digital maturity. Such studies should also test measurement invariance to determine whether the framework captures comparable constructs across countries, languages, organization types, and professional groups. This would help distinguish context-specific readiness patterns from barriers and enabling conditions that recur across humanitarian settings. Larger studies using predefined, mutually exclusive organizational and professional strata should examine whether adoption-related perceptions, organizational readiness conditions, and capacity-building priorities differ by organization type, sector, professional role, or level of seniority.

Longitudinal research would also be valuable in tracking how readiness changes over time as organizations move from early exploration toward more formalized use of AI tools. In particular, follow-up studies could examine whether improvements in governance visibility, staff capability, and organizational routines develop alongside increased experimentation and adoption. Qualitative organizational case studies would further strengthen the evidence base by exploring how leadership, internal communication, operational culture, and accountability processes shape the day-to-day use of AI in practice.

Future research should also move beyond staff and organizational perspectives alone. Humanitarian AI research will be stronger if it includes the views of crisis-affected communities and examines how AI-supported practices are experienced by those most directly affected. Responsible AI in humanitarian contexts must ultimately be assessed not only in terms of organizational readiness or operational efficiency, but also in relation to rights, trust, protection, and meaningful benefit for affected populations (20).

## 5 Conclusion

This study offers an exploratory stakeholder-based snapshot of AI readiness within Qatar’s humanitarian and development ecosystem. In this sample, respondents reported high individual openness and readiness to use AI, alongside more variable perceptions of organizational preparedness, particularly in relation to governance, oversight, and workforce capacity. These findings should be interpreted as descriptive and context-specific, but they suggest that, in this setting, practical safeguards and capacity-building may need to develop alongside growing staff interest in AI. Theoretically, the study demonstrates the value of integrating UTAUT-informed adoption perceptions with ODTR-based organizational conditions, conceptualizing responsible AI readiness as a layered construct rather than a single measure of technological preparedness. Practically, the findings suggest that humanitarian organizations, policymakers, funders, and coordinating bodies should complement AI experimentation with visible governance arrangements, role-specific workforce development, responsible-data safeguards, approved-use guidance, and mechanisms for human oversight and accountability. Future work using larger and more systematic samples, longitudinal designs, and qualitative case studies will be important to examine how readiness evolves and how responsible AI practices are operationalized across organizations.

## Data Availability

The original contributions presented in the study are included in the article/Supplementary material, further inquiries can be directed to the corresponding author.
