# Supplementary material for: Responsible AI readiness in a humanitarian and development ecosystem: an exploratory case study
Source: Front Public Health. 2026 Sep 1;14:1937518. doi: 10.3389/fpubh.2026.1937518 (PMC13576051; doi:10.3389/fpubh.2026.1937518)
Supplement: Supplementary file 1 [file Table_1.DOCX]

**Section A – Demographics & Organisational Role / القسم أ – البيانات الديموغرافية والدور في المؤسسة**

**A1.** Year of birth (YYYY)

سنة الميلاد (....)

**A2.** Gender (select one): 1 Female  2 Male

النوع الاجتماعي (اختر واحداً): 1 أنثى   2 ذكر

**A3.** Highest degree completed (1. Less than Secondary 2.Secondary or Diploma  2. Bachelor  3.Master  4. PhD/MD   5. other:)

أعلى مؤهل دراسي حصلت عليه:

1 اقل من الثانوي 2 ثانوي أو دبلوم  2 بكالوريوس  3 ماجستير  4 دكتوراه/طب  5 أخرى:

**A4.** Organisation type:

1 Government  2 Local Non Governmental Organization , University,   Organization  4 United National/IO  5 Other: ___

نوع المؤسسة:

1 جهة حكومية

2 منظمة محلية غير حكومية

3 جامعة / مؤسسة أكاديمية

4 وكالة تابعة للأمم المتحدة / منظمة دولية

5 أخرى، يُذكر: ___)

**A5.** Sector:

Health, Education, Water, Sanitation & Hygiene (WASH), Protection, Other specify

القطاع:

الصحة، التعليم، المياه والصرف والنظافة الصحية (WASH)، الحماية، أخرى (يُذكر)

**A6.** Job title / function (check all that apply):

المسمى الوظيفي / الدور (ضع علامة على كل ما ينطبق):

Executive/Director, Programme/Field Ops, Data/IT, Monitoring &Evaluation /Research, Finance/Admin, Other: ___

الإدارة العليا / المدير، البرامج / العمليات الميدانية، البيانات / تكنولوجيا المعلومات، المتابعة والتقييم / البحث، المالية / الشؤون الإدارية، أخرى: ___

**A7.** Years with current organisation

1 Less than 1  2 1-3  3 4-6  4 More than 7

عدد سنوات العمل في المؤسسة الحالية:

1 أقل من سنة  2 من -–3 سنوات  3 من- 4–6 سنوات  4 7 سنوات فأكثر

**Section B – Current Use of Technology & Data**

**القسم ب – استخدام التقنيات والبيانات حالياً**

**B1.** Have you ever worked on an AI or data-science project?

هل سبق أن عملت في مشروع يتعلق بالذكاء الاصطناعي أو علم البيانات؟

(1 Yes  2 No)

(1 نعم  2 لا)

**القسم ب – استخدام التقنيات والبيانات حالياً**

**B2.** How often does your work involve entering analyzing, or reviewing data into digital tools (e.g., Excel, Kobo, DHIS2, internal databases)?

كم مرة يتضمن عملك إدخال، مراجعة أو تحليل بيانات في أدوات رقمية (مثل إكسل، كُوبو، DHIS2 أو قواعد بيانات داخلية)؟

Never – Sometimes – Always

أبداً – أحياناً –دائماً

**B3.** I feel comfortable using digital data.

أشعر بالارتياح في استخدام البيانات الرقمية.

Disagree – Agree – Not Applicable

أعارض – أوافق – لا ينطبق

**B4.** Which types of programs managing data do you most often work with? (Select all that apply)

ما أنواع البيانات التي تعمل معها غالباً؟ (اختر كل ما ينطبق)

☐ بيانات تسجيل المستفيدين أو إدارة الحالات

Beneficiary registration or case management data, Programme monitoring data (indicators, output tracking, etc.), Financial or budget data, Logistics or supply chain data, Health or clinical data, Education or learning data, Social media or community feedback data, Other ___, Not applicable / I don’t know

بيانات متابعة البرامج (المؤشرات، تتبع المخرجات، إلخ)، بيانات مالية أو ميزانية، بيانات لوجستية أو سلسلة الإمداد، بيانات صحية أو سريرية، بيانات تعليمية أو تعلمية، بيانات من وسائل التواصل الاجتماعي أو ملاحظات المجتمع، أخرى: ______ غير منطبق / لا أعلم

**B5.** We store beneficiary data in a secure, central database.

نقوم بتخزين بيانات المستفيدين في قاعدة بيانات مركزية وآمنة.

أوافق – أعارض – لا أعلم

Agree – Disagree – I do not know

**B6.** Do you have within your organisation an ethical framework or policy that protects data?

هل لدى مؤسستك إطار أخلاقي أو سياسة تحمي البيانات (مثل سياسة حماية البيانات أو الخصوصية)؟

Yes, No, I am not sure/ I don’t know

نعم، لا، لا أعلم

**B7.** Do you collect any real-time data (e.g., social media, mobile surveys, sensors)?

هل تقومون بجمع أي بيانات آنية / لحظية (مثل بيانات من وسائل التواصل الاجتماعي، استبيانات عبر الهاتف المحمول، أجهزة استشعار)؟

☐ نعم (إذا نعم، يُرجى التوضيح: ______)

☐ Yes (If yes, please specify: ______)

☐ لا

☐ No

**Section C – AI Awareness & Perception**

**القسم ج – الوعي بالذكاء الاصطناعي والانطباعات عنه**

**C1.** I am confident that I can define what artificial intelligence (AI) means in our sector.

أنا واثق/واثقة من قدرتي على تعريف معنى الذكاء الاصطناعي (AI) في قطاعنا.

Disagree – Neutral – Agree – Not applicable / I don’t know

أعارض – محايد – أوافق – غير منطبق / لا أعلم

**C2.** I am confident that I can use AI tools in my work

Disagree – Neutral – Agree – Not applicable / I don’t know

أعارض – محايد – أوافق – غير منطبق / لا أعلم

**C3.** In your organisation, end users were involved in at least one of the AI tool development cycle (inception, designing, testing, evaluation).

في مؤسستك، تم إشراك المستخدمين النهائيين (المستفيدين أو العاملين الميدانيين) في واحدة على الأقل من مراحل تطوير أداة تعتمد على الذكاء الاصطناعي (مرحلة الفكرة، التصميم، الاختبار، التقييم).

Yes – No – I do not know

نعم – لا – لا أعلم

**C4.** Please give an example of AI used in your field (short answer).

يرجى إعطاء مثال على استخدام للذكاء الاصطناعي في مجال عملك (إجابة قصيرة).

**C5.** Which humanitarian areas might benefit from AI use? Please list all the options (e.g., logistics, data management, data collection, etc.).

ما هي مجالات العمل الإنساني التي قد تستفيد من استخدام الذكاء الاصطناعي؟ يرجى ذكر جميع المجالات الممكنة (مثل: اللوجستيات، إدارة البيانات، جمع البيانات، إلخ).

Logistics & supply-chain, Needs assessment & targeting, Data collection & management, Monitoring & evaluation (MEAL), Health services, Education & learning, Community engagement & feedback, Protection & safeguarding, Other:______

اللوجستيات وسلسلة الإمداد، تقييم الاحتياجات واستهداف المستفيدين، جمع البيانات وإدارتها، المتابعة والتقييم (MEAL)، الخدمات الصحية، التعليم والتعلّم، مشاركة المجتمع وتلقّي الملاحظات، الحماية وصون السلامة، أخرى:______

**C6.** What concerns or challenges do you have that might hinder you from using AI in humanitarian work? (Select all that apply) (merge with barriers)

ما هي المخاوف أو التحديات التي قد تمنعك من استخدام الذكاء الاصطناعي في العمل الإنساني؟ (اختر كل ما ينطبق)

☐ Lack of contextual/local data, Lack of Arabic language models

☐ نقص البيانات المحلية أو السياقية

☐ نقص نماذج اللغة العربية للذكاء الاصطناعي

☐ Data privacy breaches

☐ خروقات / انتهاكات خصوصية البيانات

☐ Cost

☐ التكلفة

☐ Complexity

☐ التعقيد الفني أو صعوبة الاستخدام

☐ Regulation uncertainty

☐ عدم وضوح الأطر التنظيمية والقانونية

☐ Lack of infrastructure in the targeted areas (electricity, smart phones, connectivity, etc.)

☐ نقص البنية التحتية في المناطق المستهدفة (الكهرباء، الهواتف الذكية، الاتصال بالإنترنت، إلخ)

☐ Other: ______

☐ أخرى: ______

**C7.** My organisation is open to use AI in its work.

منظمتي منفتحة على استخدام الذكاء الاصطناعي في عملها.

Disagree – Neutral – Agree – Not applicable / I don’t know

أعارض – محايد – أوافق – غير منطبق / لا أعلم

**Section D – Skills & Training Needs**

**القسم د – المهارات واحتياجات التدريب (سهولة الاستخدام المتصوَّرة)**

For each domain below, rate **BOTH** your current competence **and** the importance of further training.

لكل مجال من المجالات التالية، قيِّم **مستوى كفاءتك الحالية** و**أهمية الحصول على تدريب إضافي** في هذا المجال.

Scale: Limited – Fair – High

المقياس: محدود – مقبول – عالٍ

**Domain 1:** Data cleaning & visualisation

**المجال 1:** تنقية البيانات وعرضها بصرياً

D11. Competence

D11. مستوى الكفاءة

D21. Importance

D21. أهمية التدريب الإضافي

**Domain 2:** Machine-learning concepts

المجال 2: مفاهيم التعلّم الآلي

D12. Competence

D12. مستوى الكفاءة

D22. Importance

D22. أهمية التدريب الإضافي

**Domain 3:** Responsible-AI ethics

المجال 3: أخلاقيات الذكاء الاصطناعي المسؤول

D13. Competence

D13. مستوى الكفاءة

D23. Importance

D23. أهمية التدريب الإضافي

**Domain 4:** Data privacy & security

المجال 4: خصوصية البيانات وأمنها

D14. Competence

D14. مستوى الكفاءة

D24. Importance

D24. أهمية التدريب الإضافي

**Domain 5:** Gender & inclusion in tech

المجال 5: النوع الاجتماعي والشمول في التكنولوجيا

D15. Competence

D15. مستوى الكفاءة

D25. Importance

D25. أهمية التدريب الإضافي

**Domain 6:** Generative AI

**المجال 6:** الذكاء الاصطناعي التوليدي

D16. Competence

D16. مستوى الكفاءة

D26. Importance

D26. أهمية التدريب الإضافي

**Domain 7**: Data generation or data acquisition

**المجال 7:** توليد البيانات أو جمع البيانات

D17. Competence

D17. مستوى الكفاءة

D27. Importance

D27. أهمية التدريب الإضافي

**Domain 8:** Digital humanitarianism

**المجال 8:** العمل الإنساني الرقمي

D18. Competence

D18. مستوى الكفاءة

D28. Importance

D28. أهمية التدريب الإضافي

**Domain 9:** Prompt engineering

**المجال 9:** هندسة الأوامر

D19. Competence

D19. مستوى الكفاءة

D29. Importance

D29. أهمية التدريب الإضافي

**Section E – Barriers – Organisational & Environmental Readiness**

**القسم هـ – العوائق – الجاهزية المؤسسية والبيئية**

For each item, please rate how much it is a barrier in your organisation.

يرجى تقييم مدى كون كل عامل من العوامل التالية عائقاً في مؤسستك.

Scale (for E1–E6):

1 Not a barrier – Medium barrier – Major barrier

1 ليس عائقاً – عائق متوسط – عائق كبير

**E1.** Lack of budget for AI or digital projects

نقص الميزانية المخصَّصة لمشروعات الذكاء الاصطناعي أو المشروعات الرقمية

(1 Not a barrier – Medium barrier – Major barrier)

(1 ليس عائقاً – عائق متوسط – عائق كبير)

**E2.** Limited internet connectivity or hardware (computers, smartphones, etc.)

ضعف الاتصال بالإنترنت أو نقص الأجهزة (حواسيب، هواتف ذكية، إلخ)

(Not a barrier – Medium barrier – Major barrier)

(ليس عائقاً – عائق متوسط – عائق كبير)

**E3.** Insufficient staff skills to work with data or AI tools

نقص مهارات الموظفين في العمل مع البيانات أو أدوات الذكاء الاصطناعي

(Not a barrier – Medium barrier – Major barrier)

(ليس عائقاً – عائق متوسط – عائق كبير)

**E4.** Leadership resistance or lack of support for AI

مقاومة أو عدم دعم من القيادة/الإدارة لاستخدام الذكاء الاصطناعي

(Not a barrier – Medium barrier – Major barrier)

(ليس عائقاً – عائق متوسط – عائق كبير)

**E5.** Ethical or regulatory uncertainty (e.g., unclear policies, lack of guidance)

عدم وضوح الجوانب الأخلاقية أو التنظيمية (مثل غياب السياسات أو الإرشادات الواضحة)

(Not a barrier – Medium barrier – Major barrier)

(ليس عائقاً – عائق متوسط – عائق كبير)

**E6.** Community trust concerns (fear of misunderstanding or misuse of AI)

مخاوف تتعلق بثقة المجتمع (مثل الخوف من سوء فهم أو إساءة استخدام الذكاء الاصطناعي)

(Not a barrier – Medium barrier – Major barrier)

(ليس عائقاً – عائق متوسط – عائق كبير)

**E7.** Lack of contextual/local data and/or lack of Arabic AI language models

نقص البيانات المحلية أو السياقية و/أو نقص نماذج اللغة العربية للذكاء الاصطناعي

(Not a barrier – Medium barrier – Major barrier)

(ليس عائقاً – عائق متوسط – عائق كبير)

**E8.** Concerns about data privacy or potential data breaches

( Not a barrier – Medium barrier – Major barrier)

(ليس عائقاً – عائق متوسط – عائق كبير)

**E9.** Technical complexity or difficulty of using AI tools in practice

( Not a barrier – Medium barrier – Major barrier)

(ليس عائقاً – عائق متوسط – عائق كبير)

**E10. Open text:** Any other barrier or enabler we should consider?

نص مفتوح: هل هناك أي عوائق أخرى أو عوامل مساعدة ترين/ترى أنه يجب أخذها في الاعتبار؟

**Section F – Policy & Governance, Organisational Structures & Leadership Readiness**

القسم و – السياسات والحوكمة، الهياكل المؤسسية وجاهزية القيادة

**F1.** We have written SOPs covering privacy and fair data use policies.

لدينا إجراءات تشغيل مكتوبة (SOPs) تغطي سياسات الخصوصية والاستخدام العادل للبيانات.

Disagree – Neutral – Agree – Not applicable / I don’t know

أعارض – محايد – أوافق – غير منطبق / لا أعلم

**F2**. Does your organisation have a data-sharing MoU with external partners to work on AI tools?

هل لدى مؤسستك مذكرة تفاهم لمشاركة البيانات مع شركاء خارجيين للعمل على أدوات أو مشروعات ذكاء اصطناعي؟

1 Yes – 2 No – 3 I don’t know

1 نعم – 2 لا – 3 لا أعلم

**F3.** An independent ethics board reviews our digital or AI-related projects.

تقوم لجنة أخلاقيات مستقلة بمراجعة مشاريعنا الرقمية أو المشاريع المرتبطة بالذكاء الاصطناعي.

1 Yes – 2 No – 3 I don’t know

1 نعم – 2 لا – 3 لا أعلم

مفتوح: ما أهم تغيير في السياسات تعتقد أنه سيسرّع تبنّي الذكاء الاصطناعي الأخلاقي في قطر؟

**F4a.** Has your organisation developed or adapted any AI ethics framework or principles?

هل طوّرت منظمتك أو تبنّت أي إطار أو مبادئ لأخلاقيات الذكاء الاصطناعي؟

Yes – No – Other: ______

نعم – لا – أخرى: ______

**F4b.** If no, is your organisation planning or intending to develop or adopt one?

إذا كانت الإجابة لا، هل تخطّط منظمتك لتطوير أو تبنّي إطار لأخلاقيات الذكاء الاصطناعي؟

Yes – No – I don’t know

نعم – لا – لا أعلم

**F5.** Is your organisation intending to use more AI in its work in the coming years?

هل تنوي منظمتك استخدام المزيد من الذكاء الاصطناعي في عملها خلال السنوات القادمة؟

Yes – No – I don’t know

نعم – لا – لا أعلم

**F6.** Are you ready to use AI in your work?

هل أنت مستعد لاستخدام الذكاء الاصطناعي في عملك؟

Yes – No – I don’t know

نعم – لا – لا أعلم

| **Construct (TAM)** | **Question codes** | **Question text** |
| --- | --- | --- |
| **Perceived Ease of Use (PEOU)** | B3 | I feel comfortable using digital data. |
|  | B4 | Which types of programs managing data do you most often work with? (Select all that apply) |
| **Perceived Usefulness (PU)** | C5 | Which humanitarian areas might benefit from AI use? Please list all the options (e.g., logistics, data management, data collection, etc.) |
|  | D21 – D29 | Importance of 9 domains |
| **Attitude toward AI** | C1 | I am confident that I can define what artificial intelligence (AI) means in our sector. |
|  | C2 | I am confident that I can use AI tools in my work |
| **Behavioural Intention (BI)** | C6 | My organisation is open to use AI in its work. |
|  | F5 | Is your organisation intending to use more AI in its work in the coming years? |
|  | F6 | Are you ready to use AI in your work? |

| **ODTR Dimensions** | **Question codes** | **Question text** |
| --- | --- | --- |
| **Technological Resources** | B2 | How often does your work involve entering analyzing, or reviewing data into digital tools (e.g., Excel, Kobo, DHIS2, internal databases)? |
|  | B5 | We store beneficiary data in a secure, central database |
|  | C4 | Please give an example of AI used in your field (short answer). |
|  | E1 | Lack of budget for AI or digital projects |
|  | E2 | Limited internet connectivity or hardware (computers, smartphones, etc.) |
|  | E7 | Lack of contextual/local data and/or lack of Arabic AI language models |
|  | E9 | Technical complexity or difficulty of using AI tools in practice |
| **Management Capability** | B6 | Do you have within your organisation an ethical framework or policy that protects data? |
|  | B7 | Do you collect any real-time data (e.g., social media, mobile surveys, sensors)? |
|  | F1 | We have written SOPs covering privacy and fair data use policies. |
|  | F2 | Does your organisation have a data-sharing MoU with external partners to work on AI tools? |
|  | F3 | An independent ethics board reviews our digital or AI-related projects. |
| **Human Capability** | B1 | Have you ever worked on an AI or data-science project? |
|  | D11 – D19 | Competence in 9 domains |
|  | E3 | Insufficient staff skills to work with data or AI tools |
|  | E4 | Leadership resistance or lack of support for AI |
| **Corporate Culture** | C3 | In your organisation, end users were involved in at least one of the AI tool development cycle (inception, designing, testing, evaluation). |
|  | C8 | Concerns about data privacy or potential data breaches |
|  | E5 | Ethical or regulatory uncertainty (e.g., unclear policies, lack of guidance) |
|  | E6 | Community trust concerns (fear of misunderstanding or misuse of AI) |
|  | F4 | Has your organisation developed or adapted any AI ethics framework or principles? |

**Performance Expectancy (PE)**

- **C5** (Which humanitarian areas might benefit from AI use?)

**Effort Expectancy (EE)**

- **B3** (I feel comfortable using digital data.)
- **C2** (I am confident that I can use AI tools in my work.)

**Social Influence (SI)**

- **C7** (My organisation is open to use AI in its work.)

**Facilitating Conditions (FC)**

- **B5** (We store beneficiary data in a secure, central database.)

**Behavioural Intention (BI)**

- **F6** (Are you ready to use AI in your work?)

**ODTR (organisational digital/AI readiness items)**

**Technological Resources**

- **B2** (How often work involves entering/analyzing/reviewing data in digital tools)
- **B4** (Types of programs managing data you work with)
- **B7** (Collect real-time data?)
- **E1** (Lack of budget)
- **E2** (Limited connectivity/hardware)
- **E7** (Lack of contextual/local data and/or Arabic AI language models)
- **E9** (Technical complexity/difficulty using AI tools)

**Human Capability**

- **B1** (Worked on AI/data-science project?)
- **C1** (Confident defining AI in our sector)
- **C4** (Example of AI used in your field – open text)
- **D11–D19** (Competence across 9 domains)
- **D21–D29** (Importance of training across 9 domains)
- **E3** (Insufficient staff skills)

**Management Capability**

- **B6** (Ethical framework/policy that protects data?)
- **F1** (Written SOPs: privacy/fair data use)
- **F2** (Data-sharing MoU?)
- **F3** (Independent ethics board review?)
- **F4a / F4b** (AI ethics framework adopted? / planning to adopt?)
- **F5** (Organisation intending to use more AI in coming years?)
- **E4** (Leadership resistance/lack of support)
- **E5** (Ethical/regulatory uncertainty)
- **E8** (Concerns about data privacy/potential breaches)

**Corporate Culture**

- **C3** (End users involved in AI tool development cycle)
- **C6** (Concerns/challenges hindering AI use – multi-select)
- **E6** (Community trust concerns)
- **E10** (Other barrier/enabler – open text)
