# Supplementary material for: Responsible AI readiness in a humanitarian and development ecosystem: an exploratory case study
Source: Front Public Health. 2026 Sep 1;14:1937518. doi: 10.3389/fpubh.2026.1937518 (PMC13576051; doi:10.3389/fpubh.2026.1937518)
Supplement: Supplementary file 2 [file Table_3.DOCX]

Per-question summary (N=83)

======================================================================

Q2

Q2_Year of birth

Missing: 0/83

Open-ended responses: 83/83

Q3

Q3_Gender

Missing: 0/83

- Male: 54 (65.1%)

- Female: 29 (34.9%)

Q4

Q4_Highest degree completed

Missing: 0/83

- Master: 45 (54.2%)

- Bachelor: 29 (34.9%)

- PhD/MD: 7 (8.4%)

- Other: 2 (2.4%)

Q5

Q5_Organisation type

Missing: 0/83

- United National/ International Organization: 35 (42.2%)

- Governmental: 21 (25.3%)

- Local Non-Governmental Organization: 20 (24.1%)

- University/ Academic: 4 (4.8%)

- Other: 3 (3.6%)

Q6

Q6_Sector

Missing: 0/83

- Other: 33 (39.8%)

- Health: 27 (32.5%)

- Water, Sanitation & Hygiene (WASH): 13 (15.7%)

- Education: 9 (10.8%)

- Protection: 1 (1.2%)

Q7

Q7_Job title / function (check all that apply)

Missing: 0/83

- Programme/Field Ops: 28 (33.7%)

- Executive/Director: 24 (28.9%)

- Other: 14 (16.9%)

- Monitoring &Evaluation /Research: 10 (12.0%)

- Data/IT: 4 (4.8%)

- Finance/Admin: 3 (3.6%)

Q8

Q8_Years with current organisation

Missing: 0/83

- More than 7 years: 35 (42.2%)

- 1 to 3 years: 21 (25.3%)

- 4 to 6 years: 15 (18.1%)

- Less than 1 year: 12 (14.5%)

Q9

Q9_Have you ever worked on an AI or data-science project?

Missing: 0/83

- No: 45 (54.2%)

- Yes: 38 (45.8%)

Q10

Q10_How often does your work involve entering analyzing, or reviewing data into digital tools (e.g., Excel, Kobo, DHIS2, internal databases

Missing: 0/83

- Always: 53 (63.9%)

- Sometimes: 29 (34.9%)

- Never: 1 (1.2%)

Q11

Q11_I feel comfortable using digital data.

Missing: 0/83

- Agree: 73 (88.0%)

- Disagree: 7 (8.4%)

- Not Applicable: 3 (3.6%)

Q12

Q12_Which types of programs managing data do you most often work with? (Select all that apply)

Missing: 0/83

- Programme monitoring data (indicators: 42 (50.6%)

- output tracking: 42 (50.6%)

- etc.): 42 (50.6%)

- Beneficiary registration or case management data: 31 (37.3%)

- Financial or budget data: 31 (37.3%)

- Health or clinical data: 21 (25.3%)

- Logistics or supply chain data: 20 (24.1%)

- Education or learning data: 16 (19.3%)

- Social media or community feedback data: 12 (14.5%)

Q13

Q13_We store beneficiary data in a secure, central database.

Missing: 0/83

- Agree: 58 (69.9%)

- I do not know: 13 (15.7%)

- Disagree: 12 (14.5%)

Q14

Q14_Do you have within your organisation an ethical framework or policy that protects data?

Missing: 0/83

- Yes: 62 (74.7%)

- I do not know: 16 (19.3%)

- No: 5 (6.0%)

Q15

Q15_Do you collect any real-time data (e.g., social media, mobile surveys, sensors)?

Missing: 0/83

- No: 56 (67.5%)

- Yes: 27 (32.5%)

Q16

Q16_I am confident that I can define what artificial intelligence (AI) means in our sector

Missing: 0/83

- Agree: 66 (79.5%)

- Not applicable / I don’t know: 10 (12.0%)

- Disagree: 7 (8.4%)

Q17

Q17_I am confident that I can use AI tools in my work

Missing: 0/83

- Agree: 66 (79.5%)

- Not applicable / I don’t know: 9 (10.8%)

- Disagree: 8 (9.6%)

Q18

Q18_In your organisation, end users were involved in at least one of the AI tool development cycle (inception, designing, testing, evaluation)

Missing: 0/83

- Yes: 37 (44.6%)

- No: 25 (30.1%)

- I do not know: 21 (25.3%)

Q19

Q19_Please give an example of AI used in your field (not necessarily in your own organization

Missing: 1/83

Open-ended responses: 82/83

Q20

Q20_Which humanitarian areas might benefit from AI use? Please list all the options (e.g., logistics, data management, data collection, etc.).

Missing: 0/83

- Data collection & management: 69 (83.1%)

- Needs assessment & targeting: 69 (83.1%)

- Monitoring & evaluation (MEAL): 64 (77.1%)

- Logistics & supply-chain: 52 (62.7%)

- Education & learning: 50 (60.2%)

- Community engagement & feedback: 49 (59.0%)

- Health services: 42 (50.6%)

- Protection & safeguarding: 38 (45.8%)

- Other: 3 (3.6%)

Q21

Q21_My organisation is open to use AI in its work

Missing: 0/83

- Agree: 68 (81.9%)

- Not applicable / I don’t know: 11 (13.3%)

- Disagree: 4 (4.8%)

Q22

Q22_Domain 1: Data cleaning & visualisation_Competence

Missing: 0/83

- High: 51 (61.4%)

- Fair: 44 (53.0%)

- Fair: 27 (32.5%)

- Limited: 22 (26.5%)

- High: 17 (20.5%)

- Limited: 5 (6.0%)

Q23

Q23_Domain 2: Machine-learning concepts_Competence

Missing: 0/83

- Fair: 39 (47.0%)

- Fair: 38 (45.8%)

- Limited: 33 (39.8%)

- High: 33 (39.8%)

- High: 12 (14.5%)

- Limited: 11 (13.3%)

Q24

Q24_Domain 3: Responsible-AI ethics_Competence

Missing: 0/83

- High: 51 (61.4%)

- Fair: 40 (48.2%)

- Fair: 24 (28.9%)

- High: 23 (27.7%)

- Limited: 20 (24.1%)

- Limited: 8 (9.6%)

Q25

Q25_Domain 4: Data privacy & security_Competence

Missing: 0/83

- High: 58 (69.9%)

- High: 35 (42.2%)

- Fair: 29 (34.9%)

- Limited: 19 (22.9%)

- Fair: 18 (21.7%)

- Limited: 7 (8.4%)

Q26

Q26_Domain 5: Gender & inclusion in tech_Competence

Missing: 0/83

- High: 38 (45.8%)

- Fair: 32 (38.6%)

- Fair: 32 (38.6%)

- Limited: 29 (34.9%)

- High: 22 (26.5%)

- Limited: 13 (15.7%)

Q27

Q27_Domain 6: Generative AI_Competence

Missing: 0/83

- High: 37 (44.6%)

- Fair: 37 (44.6%)

- Fair: 35 (42.2%)

- Limited: 31 (37.3%)

- High: 17 (20.5%)

- Limited: 9 (10.8%)

Q28

Q28_Domain 7: Data Generation or Data Acquisition_Competence

Missing: 0/83

- High: 38 (45.8%)

- Fair: 34 (41.0%)

- Fair: 33 (39.8%)

- Limited: 28 (33.7%)

- High: 21 (25.3%)

- Limited: 12 (14.5%)

Q29

Q29_Domain 8: Digital humanitarianism_Competence

Missing: 0/83

- High: 52 (62.7%)

- Limited: 35 (42.2%)

- Fair: 27 (32.5%)

- High: 21 (25.3%)

- Fair: 20 (24.1%)

- Limited: 11 (13.3%)

Q30

Q30_Domain 9: Prompt engineering_Competence

Missing: 0/83

- High: 37 (44.6%)

- Fair: 36 (43.4%)

- Limited: 32 (38.6%)

- Fair: 27 (32.5%)

- Limited: 19 (22.9%)

- High: 15 (18.1%)

Q31

Q31_For each item, please rate how much it is a barrier in your organisation._Lack of budget for AI or digital projects

Missing: 0/83

- Medium barrier: 50 (60.2%)

- Medium barrier: 47 (56.6%)

- Medium barrier: 45 (54.2%)

- Not a barrier: 44 (53.0%)

- Medium barrier: 44 (53.0%)

- Medium barrier: 40 (48.2%)

- Medium barrier: 37 (44.6%)

- Medium barrier: 37 (44.6%)

- Not a barrier: 34 (41.0%)

- Medium barrier: 34 (41.0%)

- Major barrier: 33 (39.8%)

- Medium barrier: 31 (37.3%)

- Major barrier: 30 (36.1%)

- Major barrier: 27 (32.5%)

- Major barrier: 25 (30.1%)

- Major barrier: 25 (30.1%)

- Major barrier: 21 (25.3%)

- Not a barrier: 19 (22.9%)

- Major barrier: 19 (22.9%)

- Not a barrier: 18 (21.7%)

- Not a barrier: 17 (20.5%)

- Major barrier: 15 (18.1%)

- Not a barrier: 14 (16.9%)

- Not a barrier: 13 (15.7%)

- Not a barrier: 11 (13.3%)

- Not a barrier: 9 (10.8%)

- Major barrier: 8 (9.6%)

Q32

Q32_Any other barrier or enabler we should consider?

Missing: 10/83

Open-ended responses: 73/83

Q33

Q33_We have written SOPs covering privacy and fair data use policies

Missing: 0/83

- Agree: 44 (53.0%)

- Not applicable / I don’t know: 27 (32.5%)

- Disagree: 12 (14.5%)

Q34

Q34_Does your organisation have a data-sharing MoU with external partners to work on AI tools?

Missing: 0/83

- I do not know: 37 (44.6%)

- No: 25 (30.1%)

- Agree: 21 (25.3%)

Q35

Q35_An independent ethics board reviews our digital or AI-related projects.

Missing: 0/83

- I don’t know: 40 (48.2%)

- No: 28 (33.7%)

- Yes: 15 (18.1%)

Q36

Q36_Has your organisation developed or adapted any AI ethics framework or principles? If no, is your organisation planning or intending to develop or adopt one? (Reply with Yes, No, I do not know)

Missing: 0/83

- I do not know: 47 (56.6%)

- Yes: 23 (27.7%)

- No: 13 (15.7%)

Q37

Q37_Is your organisation intending to use more AI in its work in the coming years?

Missing: 0/83

- Yes: 50 (60.2%)

- I do not know: 30 (36.1%)

- No: 3 (3.6%)

Q38

Q38_Are you ready to use AI in your work?

Missing: 0/83

- Yes: 76 (91.6%)

- No: 7 (8.4%)
